# Type 2 diabetes mellitus, cognition and brain in aging: A brief review

**Published:** 2009-01

**Authors:** Rajeev Kumar, Jeffrey C. L. Looi, Beverley Raphael

**Affiliations:** Academic Unit of Psychological Medicine, Australian National University Medical School, College of Medicine, Biology and Environment, Canberra Hospital, Canberra, Australia

**Keywords:** Brain atrophy, cognition, diabetes mellitus, magnetic resonance imaging, neurodegeneration

## Abstract

Diabetes mellitus is a complex disease with many potential complications. Whilst there have been inconsistent results in regard to an association between cognition and type 2 diabetes, there is evidence that verbal memory and processing speed are the cognitive domains usually impaired. In elderly diabetic subjects, other cognitive domains may also be involved, due to ageing. Glycemic control is implicated in the development of cognitive dysfunction, although more research is needed in this area. Insulin dysregulation and hyperglycemia play an important role in neurodegeneration. Using structural neuroimaging, it has been shown that brain atrophy is an important feature in those with type 2 diabetes. Integrative research is needed using behavioral, cognitive, imaging, and genetic platforms.

## INTRODUCTION

Diabetes mellitus is a complex endocrine disease that can lead to many complications particularly when untreated. The association between type 2 diabetes mellitus and dementia has been of great interest, and this is particularly relevant with the increase in prevalence of both diabetes and dementia with increasing life expectancy.[1–3] Although type 2 diabetes is associated with several other vascular risk factors of dementia, diabetes itself has been implicated as an independent risk factor for cognitive impairment and dementia.[4–7]

Neurocognitive research in diabetes mellitus has provided us with greater understanding of the relationship between brain and cognition. This has been possible mainly by the application of neuroimaging and use of comprehensive standardized neuropsychological assessments. Animal research into basic pathophysiological mechanisms has indicated possible pathways of neurodegeneration. In this brief review, we discuss some of the important findings from studies on cognition, neuroimaging, and pathophysiology in type 2 diabetes mellitus. It is known that depression may contribute to cognitive changes in diabetes, but this review concentrates on studies excluding those with depression, focusing on cognition and diabetes.

## METHODOLOGY

We conducted a selective brief review of literature. A MEDLINE search was conducted using the following medical subject heading terms: cognition, dementia, cognitive impairment, brain atrophy, white matter hyperintensities, brain imaging, magnetic resonance imaging (MRI) and pathophysiology; all combined with “diabetes”. Relevant articles were retrieved based on their importance from reading the abstract. Important articles were also reviewed from authors’ collection.

## TYPE 2 DIABETES AND COGNITION

In a previous literature review, it was concluded that while cross-sectional studies yielded conflicting results, longitudinal studies have clearly demonstrated that diabetes mellitus is associated with increased rate of cognitive decline or an increased incidence of dementia.[4] There is ongoing debate about the nature and magnitude of cognitive dysfunction in subjects with type 2 diabetes. The cognitive domains that have been studied in type 2 diabetes include: memory, psychomotor speed, visuospatial functions, frontal executive functions, processing speed, verbal fluency, attention, and complex motor functions. In a comprehensive review of literature on the association between impaired glucose tolerance, type 2 diabetes, and cognitive function, it was concluded that the most consistently reported measures were impairment in verbal memory and processing speed, with preservation of functions in other areas including visuospatial function, attention, semantic memory, and language.[8] However, it has been argued that the preservation of memory and learning functions in some studies occurred mainly in subjects younger than 65 years of age in contrast to older subjects where impairments in those domains were largely because of interaction between diabetes-related changes and the normal ageing changes in the brain.[9] Younger subjects with type 2 diabetes mellitus consistently showed impairment in psychomotor efficiency, similar to subjects with type 1 diabetes. However, the impact of such cognitive deficits on day-to-day functioning is unclear, but in combination with depression, which is common in diabetes, may be significant. In a recent study, Bruce *et al*.[10] showed that about 17% of elderly diabetic patients had moderate to severe impairments in activities of daily living, 11% were cognitively impaired and 14% depressed.

## GLYCEMIC CONTROL AND COGNITION

Hyperglycemia, impaired glucose tolerance, and hypoglycemic episodes have been implicated as causes for impaired cognitive function in diabetic patients. Glycosylated haemoglobin (HbA1c) is a marker of glycemic control, and in a study by Yaffe *et al*.[11] an Hb1Ac of more than 7% was associated with a 4-fold increase in occurrence of mild cognitive impairment. An impairment in working memory, frontal executive functions, learning and complex psychomotor abilities have also been found associated with a higher level of HbA1c.[12–14] However, other studies failed to show such associations.[15,16] Impaired glucose tolerance, in the absence of a diagnosis of diabetes has been associated with cognitive dysfunction.[17,18] The results are inconsistent, with some studies showing negative results.[19,20] Reasons for such inconsistencies could be due to lack of adequate control of confounders such as education, comorbid medical and neurological conditions and the blood sugar level at the time of testing. Negative effects of recurrent and severe hypoglycemic episodes on cognition have been studied and the results have proved inconclusive. A detailed discussion is beyond the scope of this review as most data is based upon children and adolescents with type 1 diabetes, with limited or no data on older subjects such as those with type 2 diabetes. Acute hypoglycemia has been associated with impaired cognition.[21,22] Of the long-term effects of hypoglycemic episodes on cognition, there are some reports of an association.[23,24]

## PATHOPHYSIOLOGY OF COGNITIVE DYSFUNCTION

Proposed pathogenetic mechanisms of cognitive dysfunction in diabetes include chronic hypoglycemia, vascular disease, cumulative effect of hypoglycemic events, and possible direct effects of insulin on the brain.[25] It is unclear whether these factors individually, or in combination, mediate the pathogenesis of the cognitive dysfunction. In diabetes, there is a significant cumulative burden of cerebrovascular disease resulting from diabetic microangiopathy, hyperlipidaemia, and potentiation of other vascular risk factors such as hypertension. Another key mechanism of neurodegeneration in diabetics may be insulin dysregulation.[26] This may result in increased inflammation, oxidative stress, advanced glycation end products, decreased neuronal repair and neurogenesis. Insulin can promote the release of intracellular Ab in neuronal cultures[27] and intracellular accumulation of Ab has been considered as a pertinent factor in the pathogenesis of Alzheimer’s disease (AD).[28] These findings indicate that diabetes, through neurodegenerative processes resulting from dysfunctional metabolism, may lead to direct neuronal injury manifest as brain atrophy, and a structural basis for dementia.

## BRAIN IMAGING STUDIES

Since brain imaging provides an avenue for understanding the nature and magnitude of brain pathology associated with diseases that affect cognition, it has been utilized in several studies of cognition and diabetes.[29] Structural brain imaging has essentially investigated two aspects of brain pathology, cerebrovascular disease (white matter lesions, lacunar infarctions) and neuronal degeneration (subcortical and cortical cerebral atrophy). These studies involved subjects from both clinic[30–34] and community settings[35–38] providing interesting, but inconsistent results. Most studies have involved older subjects and observer-visual rating of white matter lesions and brain atrophy. To address this issue, we investigated this in a 60-64 year old community-dwelling sample of 39 subjects with type 2 diabetes and 428 normal comparison subjects.[39] All subjects underwent physical examinations, assessment of depression, cognitive assessments, brain magnetic resonance imaging scans (MRI), and fasting blood tests. We used brain MRI automated measures of atrophy, white matter intensities, and hippocampal volumetry. We found that diabetic subjects showed increased brain atrophy and poor motor function, independent of depression in comparison to healthy community controls. Brands *et al*.[40] from Netherlands showed similar results that subjects with type 2 diabetes were more depressed, had worse MRI ratings than controls, and poor performance on cognitive tests, however, there was no interaction between depression and cognitive disturbances. Brain atrophy has been an important finding in many studies.[38,41–43] Others have also reported hippocampal atrophy.[31,35] Thus, increased brain atrophy associated with poor motor function and cognitive dysfunction, independent of depression, have been found in those with type 2 diabetes.

## CONCLUSIONS AND FUTURE DIRECTIONS

Based on the studies discussed above, it is clear that type 2 diabetes has an effect on cognition, brain structural integrity, and function. An issue still remains is that what is the main pathophysiology that leads to cognitive impairment - is it purely degenerative, exclusively vascular or a combination of these two? More importantly, several other associated variables could also interact in patients with diabetes. These include hypertension, obesity, hypercholesterolemia, stroke, microvascular diseases, etc. Another important variable, which is understudied in the context of cognitive impairment and brain structural and functional pathology is depression. Since depression is a common problem in diabetics, this should be looked at carefully in future studies of cognitive impairment in diabetes. In an era of genetics and genomics, we should take advantage of such platforms in collaborative research involving studies of cognition and brain in diabetics, and the insights from such studies would lead to development of effective therapeutic strategies. Newer neuroimaging modalities such as diffusion tensor imaging could be useful to study very early brain changes in the white matter of diabetic patients. In our view, an integrative research approach using multiple platforms of behavioral, cognitive, imaging and genetic disciplines is the ideal way forward for future neurocognitive research in all neuropsychiatric disorders especially diabetes mellitus.
